# Impact of Natural and Human Factors on Dryland Vegetation in Eurasia from 2003 to 2022

**DOI:** 10.3390/plants13212985

**Published:** 2024-10-25

**Authors:** Jinyue Liu, Jie Zhao, Junhao He, Pengyi Zhang, Fan Yi, Chao Yue, Liang Wang, Dawei Mei, Si Teng, Luyao Duan, Nuoxi Sun, Zhenhong Hu

**Affiliations:** 1 State Key Laboratory of Soil Erosion and Dryland Farming on the Loess Plateau, Institute of Soil and Water Conservation, Northwest A&F University, Yangling 712100, Shaanxi, China; liujinyue_cn@163.com (J.L.); junhaohe@nwafu.edu.cn (J.H.); yifanyf@nwafu.edu.cn (F.Y.); chaoyuejoy@gmail.com (C.Y.); 2 Shandong Provincial Key Laboratory of Water and Soil Conservation and Environmental Protection, College of Resources and Environment, Linyi University, Linyi 276000, Shandong, China; wangliang.cn@163.com (L.W.); meidawei@lyu.edu.cn (D.M.); tengsi_ak@163.com (S.T.); duan_n151212@163.com (L.D.); nuoxi_sun@163.com (N.S.); 3 College of Natural Resources and Environment, Northwest A&F University, Yangling 712100, Shaanxi, China; zhangpengyi33@gmail.com; 4 State Key Laboratory of Soil Erosion and Dryland Farming on the Loess Plateau, Northwest A&F University, Yangling 712100, Shaanxi, China

**Keywords:** vegetation coverage change, natural change, human activities, residual trend analysis

## Abstract

Eurasian dryland ecosystems consist mainly of cropland and grassland, and their changes are driven by both natural factors and human activities. This study utilized the normalized difference vegetation index (NDVI), gross primary productivity (GPP) and solar-induced chlorophyll fluorescence (SIF) to analyze the changing characteristics of vegetation activity in Eurasia over the past two decades. Additionally, we integrated the mean annual temperature (MAT), the mean annual precipitation (MAP), the soil moisture (SM), the vapor pressure deficit (VPD) and the terrestrial water storage (TWS) to analyze natural factors’ influence on the vegetation activity from 2003 to 2022. Through partial correlation and residual analysis, we quantitatively described the contributions of both natural and human factors to changes in vegetation activity. The results indicated an overall increasing trend in vegetation activity in Eurasia; the growth rates of vegetation greenness, productivity and photosynthetic capacity were 1.00 × 10^−3^ yr^−1^ (*p* < 0.01), 1.30 g C m^−2^ yr^−2^ (*p* < 0.01) and 1.00 × 10^−3^ Wm^−2^μm^−1^sr^−1^yr^−1^ (*p* < 0.01), respectively. Furthermore, we found that soil moisture was the most important natural factor influencing vegetation activity. Human activities were identified as the main driving factors of vegetation activity in the Eurasian drylands. The relative contributions of human-induced changes to NDVI, GPP and SIF were 52.45%, 55.81% and 74.18%, respectively. These findings can deepen our understanding of the impacts of current natural change and intensified human activities on dryland vegetation coverage change in Eurasia.

## 1. Introduction

As one of the most concentrated regions of global arid and semi-arid areas, the Eurasian drylands are facing the risks of expanding arid areas and ecological degradation [1,2]. Vegetation is an integral component of dryland ecosystems, playing a crucial role in carbon sequestration, climate and supporting human activity in Eurasia [3]. Eurasian dryland ecosystems consist mainly of grasslands and croplands, which are highly susceptible to hydrothermal factors and anthropogenic disturbances [3,4]. Most countries in the Eurasian drylands are developing countries that rely mainly on agriculture and livestock for survival [5]. Therefore, clarifying the vegetation activity (grassland and cropland) and its natural and human-induced influences is crucial for protecting ecosystems, improving livelihoods and achieving sustainable development goals [6].

Monitoring vegetation activity through remote sensing and analyzing its relationships with natural changes has become a crucial research field on a global or a specific regional scale in recent years [7,8,9,10]. The normalized vegetation index (NDVI), derived from infrared and near-infrared remote sensing data, is an excellent indicator widely applied in studying vegetation activity on a global or a specific regional scale, reflecting vegetation greenness effectively [11]. However, in specific situations, such as under severe drought conditions or in areas with low vegetation cover, the accuracy of NDVI in reflecting vegetation activity is limited [12]. The gross primary productivity (GPP) of terrestrial ecosystems, signifying the total organic carbon assimilated by vegetation through photosynthesis per unit of time and area, constitutes the largest carbon flux in the carbon budget and plays a fundamental role in the carbon cycle [13]. Quantifying terrestrial gross primary productivity (GPP) and its changes is crucial for understanding the dynamic characteristics of global and regional vegetation productivity [14,15]. In addition, compared to the traditional vegetation greenness and productivity index (NDVI and GPP), solar-induced chlorophyll fluorescence (SIF) is a newly remote sensing satellite dataset and is known to be an effective probe for vegetation photosynthesis [16,17]. Many scholars have used the SIF dataset to analyze vegetation cover changes in regions [18,19,20,21]. In summary, NDVI, GPP and SIF can all, to a certain extent, reflect vegetation activity, but each indicator emphasizes different aspects. Therefore, for a comprehensive understanding of vegetation activity in the Eurasian drylands, it is necessary to conduct a comprehensive and systematic analysis using multiple vegetation-related indicators.

Hydrothermal factors are the most crucial natural indicators influencing vegetation activity [22,23,24]. Previous studies have highlighted that increased precipitation plays a significant role in promoting vegetation greening, especially in global drylands. Additionally, an increasing amount of evidence suggests that water availability, including soil moisture (SM), atmospheric vapor pressure deficit (VPD) and terrestrial water storage (TWS), plays an increasingly crucial role in determining vegetation activity over drylands [25,26,27]. Among them, VPD denotes the difference between saturation vapor pressure and the actual water vapor pressure at a given temperature [28]. Vegetation experiences water stress due to both high atmospheric water demand and limited water supply during drought events, leading to diminished vegetation growth with higher VPD [28]. A recent study highlighted that VPD had a widespread constraint on the interannual variability of GPP [29]. However, the constraints of VPD variations affecting vegetation growth in the Eurasian drylands has not been quantified. Moreover, recent research suggests that dryland vegetation may be particularly sensitive to soil moisture [30] and that TWS, which quantifies total water availability, can affect vegetation more rapidly than precipitation changes alone [31,32]. Eurasian drylands, as a region strongly regulated by water availability, are influenced by the accessibility of water, which in turn affects vegetation [6]. Therefore, variations in individual water factors alone cannot fully explain vegetation activity in drylands.

In addition to various natural factors, human activities can also influence dryland vegetation at the landscape scale [33]. For instance, the conversion of farmland to forests and grassland driven by policy initiatives, such as the “Grain for Green” program, has increased vegetation productivity in the Loess Plateau of China since 2000 [34]. Agricultural development is vigorously pursued in countries like Turkey, China and India, contributing to greening in the Eurasian drylands [35]. Additionally, neglecting local climate factors, engaging in irrational farming practices, excessive reclamation, overgrazing and urban expansion will cause a significant decline in vegetation coverage [36]. During the Soviet era, large swaths of steppe in northern Kazakhstan were converted to cropland, some of which were abandoned after the collapse of the Soviet Union [37]. However, in comparison to natural change, less attention has been given to the impact of human activities in the Eurasian drylands. The relative contribution of various natural and human factors on dryland vegetation is still not fully understood.

Therefore, the main goals of this study are to (1) quantify the spatiotemporal patterns of vegetation greenness, productivity and photosynthetic capacity from 2003 to 2022 in the Eurasian drylands; (2) determine the impact of individual natural drivers on dryland vegetation; and (3) distinguish and quantify the contributions of both natural and human factors to the inter-annual variations in dryland vegetation. We hypothesize that both natural factors and human activities significantly influence the dryland vegetation (grassland and cropland) in Eurasia, with distinct patterns observed in the responses of these ecosystems to environmental changes over the period from 2003 to 2022. We believe that understanding the natural factors can help reduce the financial investment required for ecological restoration initiatives. The findings may support the enhancement of vegetation growth, thereby aiding in ecological conservation efforts in Eurasian regions.

## 2. Materials and Methods

### 2.1. Study Area

**Figure 1:**
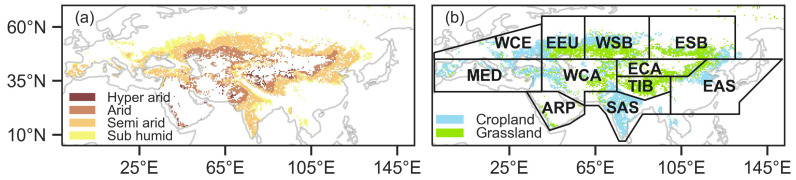
The spatial distribution map of aridity levels (**a**) and vegetation types (**b**) in the Eurasian drylands. WCE, EEU, the MED, WSB, ESB, WCA, ECA, TIB, EAS, ARP and SAS represent West and Central Europe, E. Europe, Mediterranean, W. Siberia, E. Siberia, W. C. Asia, E. C. Asia, Tibet Plateau, E. Asia, Arabian Peninsula and S. Asia, respectively.

The Eurasian drylands constitute the largest arid zone on the globe (Figure 1). Most countries in the Eurasian drylands are developing, with a high proportion of people living below the United Nations poverty line [6]. The ecosystems in the Eurasian drylands primarily consist of grassland and cropland [4]. Agriculture and livestock farming are widely practiced in this region, occurring in both mountainous areas and relatively flat grassland. Dryland is defined by the standard threshold of aridity indices (AIs) as <0.65 (dry subhumid: 0.5 ≤ AI < 0.65, semiarid: 0.2 ≤ AI < 0.5, arid: 0.05 ≤ AI < 0.2, hyperarid: AI < 0.05) (Figure 1a) [38,39] (Http://figshare.com/articles/dataset/Global_Aridity_Index-_and_Potential_Evapotranspiration_ET0_Climate_Database_v2/7504448/3?file=13901336, accessed on 10 January 2024). The eleven sub-regions of the Eurasian drylands are based on the adjustment of the partitions proposed by the Intergovernmental Panel on Climate Change (IPCC) [40] (Figure 1b).

### 2.2. Data

The NDVI and GPP data from 2003 to 2022 were extracted from the Moderate Resolution Imaging Spectroradiometer (MODIS) product (MOD13A3 and MOD17A2H) (https://lpdaac.usgs.gov/products/, accessed on 15 May 2024). The MODIS NDVI product is provided at a monthly temporal interval with a spatial resolution of 1000 m [41]. The MODIS GPP product is available with a cumulative 8-day temporal frequency and a spatial resolution of 500 m [42]. The SIF data used in this study from 2003 to 2022 were sourced from the recently released global dataset of the solar-induced chlorophyll fluorescence (GOSIF) dataset. This product is based on observations obtained by the Orbiting Carbon Observatory-2 (OCO-2), using machine learning methods to derive discrete SIF measurements. It provides 8-day SIF data at a spatial resolution of 0.05° globally [43] (https://globalecology.unh.edu/data/GOSIF.html). The monthly dataset was generated by applying the Maximum Value Composition (MVC) method to the GPP and SIF.

The monthly mean annual temperature (MAT), mean annual precipitation (MAP), AVP, Tmax and Tmin data from 2003 to 2022 were obtained from the gridded Climate Research Unit, University of East Anglia (CRU TS v4.07) dataset [30,44] (https://crudata.ue-a.ac.uk/cru/data/hrg/cru_ts_4.07/, accessed on 8 June 2024). The SM data were obtained through the ERA5 product, with a spatial resolution of 0.1°. This dataset includes four layers (0–7 cm, 7–28 cm, 28–100 cm and 100–289 cm) (https://cds.climate.copernicus.eu/cd-sapp#!/dataset/reanalysis-era5-land-monthly-means?tab=form, accessed on 12 June 2024) [45,46]. Monthly SM was calculated by weighted average. The TWS data from 2003 to 2022 were obtained from GRACE level RL06 data provided by JPL (https://grace.jpl.nasa.gov/data/get-data/jpl_global_mascons/, accessed on 12 June 2024) [47,48]. Missing months were supplemented using linear interpolation. All the variables above were resampled to a spatial resolution of 0.5° using the bilinear interpolation method. The VPD can be calculated as follow [29]:

(1) $$\begin{array}{c} \;VPD=SVP-AVP \end{array}$$

wherein SVP can be calculated based on the following formula using $T_{max}$ and $T_{min}$:

(2) $$\begin{array}{c} \;SVP=0.5\times \left(0.611\times \exp\left(\frac{17.3\times T_{min}}{T_{min}+237.3}\right)+0.611\times \exp\left(\frac{17.3\times T_{max}}{T_{max}+237.3}\right)\;\right) \end{array}$$

The land cover dataset for 2010 was obtained from the European Space Agency (ESA) Climate Change Initiative (CCI) (www.esa-landcover-cci.org/, accessed on 20 January 2024). The ESA CCI dataset, developed according to the resolution of 300 m using a classification scheme by the Food and Agriculture Organization (FAO), categorizing land cover into 37 classes (22 global categories and 15 regional categories). In this study, the original ESA CCI land cover types were reclassified into two classes, grassland and cropland (Table 1), and then resampled to a spatial resolution of 0.5° using the nearest-neighbor method.

### 2.3. Method

#### 2.3.1. Linear Trend Analysis

The trend analysis for NDVI, GPP, SIF MAT, MAP, SM, VPD and TWS utilized least squares linear regression [7]. Positive regression coefficients denoted increasing trends, while negative coefficients indicated a decreasing trend [49]. The significance of linear regression coefficients was assessed using a two-tailed *t*-test [50]. Pixel-based calculations were further employed to analyze the spatiotemporal patterns of trends in natural variables and vegetation variability in the Eurasian drylands from 2003 to 2022 [50].

#### 2.3.2. Partial Correlation Analysis

Several natural variables tested as predictors of vegetation indices (NDVI, GPP and SIF) exhibiting covariation. When detecting the relationship between the detrended natural factors and the detrended vegetation variables, it is crucial to address this covariance [51]. Partial correlation analysis is a widely utilized statistical tool for isolating the relationship between two variables from the confounding effects of many correlated variables [8,51]. In assessing the relationship between NDVI, GPP, or SIF and natural factors, we employed four-order partial correlation analyses from the “pingouin” Python package to eliminate the confounding effects of the other variables. For instance, the partial correlation coefficient between NDVI and MAT was calculated by restricting MAP, SM, VPD and TWS.

#### 2.3.3. Residual Trend Analysis

The residual trend analysis method, based on the strong correlation between vegetation and natural factors, can be used to distinguish human-induced vegetation from the effects arising from natural factors [52]. This approach assumes that by removing natural factor signals from vegetation activity, the residuals can be attributed to human influences [52]. In this study, we established a multiple linear regression model among natural factors and vegetation activity variables to separate the impacts of human-induced vegetation activity from those of natural factors [53]. Taking NDVI as an example, the difference between the predicted values (NDVI_pre_) and the observed values (NDVI_obs_) is the residual values (NDVI_res_), reflecting the response of vegetation to human activities [54,55]:

(3) $$\begin{array}{c} \;y_{pre}=a\times MAT+b\times MAP+c\times SM+d\times VPD+e\times TWS+f\; \end{array}$$

(4) $$\begin{array}{c} \;y_{res}=y_{obs}+y_{pre}\; \end{array}$$

where y represents the dependent variable, a, b, c, d and e represent the regression coefficients and f represents the constants.

(5) $$\begin{array}{c} \;Slope=\frac{n\times \sum_{i=1}^{n}\left(i\times x_{i}\right)-\sum_{i=1}^{n}i\sum_{i=1}^{n}\left(x_{i}\right)}{n\times \sum_{i=1}^{n}i^{2}-{\left(\sum_{i=1}^{n}i\right)}^{2}}\; \end{array}$$

where n represents the year. The slope represents the trend in the NDVI, GPP and SIF during 2003–2022.

The relative contributions of natural variables and human activities in vegetation change were also calculated (Table 2).

Given that GRACE began collecting data in April 2002, the time frame for our study was set from 2003 to 2022 to ensure a complete annual dataset. All the statistical analyses were performed using Python version 3.8.13.

## 3. Results

### 3.1. Spatiotemporal Vegetation Activities in Eurasian Drylands

**Figure 2:**
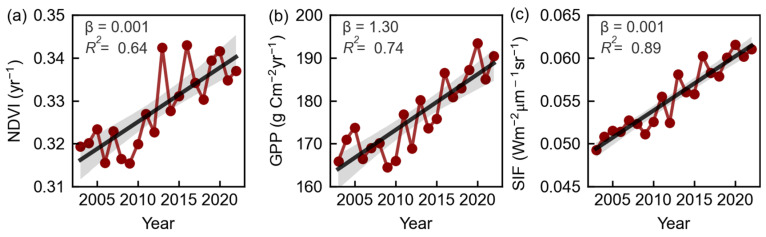
Interannual variation of normalized difference vegetation index (NDVI, (**a**)), gross primary productivity (GPP, (**b**)) and solar-induced chlorophyll fluorescence (SIF, (**c**)) in Eurasian drylands during 2003–2022. Shading denotes 95% prediction intervals. All regressions were significant (*p* < 0.05, the Student’s *t*-test).

Between 2003 and 2022, the growth rates of the normalized vegetation index (NDVI), gross primary productivity (GPP) and solar-induced chlorophyll fluorescence (SIF) in the Eurasian drylands were 1.00 × 10^−3^ yr^−1^ (*p* < 0.01), 1.30 g C m^−2^ yr^−2^ (*p* < 0.01) and 1.00 × 10^−3^ Wm^−2^µm^−1^sr^−1^yr^−1^ (*p* < 0.01), respectively (Figure 2). This suggests a significant increase in vegetation greenness, productivity and photosynthetic capacity in the Eurasian drylands. In other words, over the past two decades, the Eurasian drylands have exhibited an increasing trend in vegetation activity.

**Figure 3:**
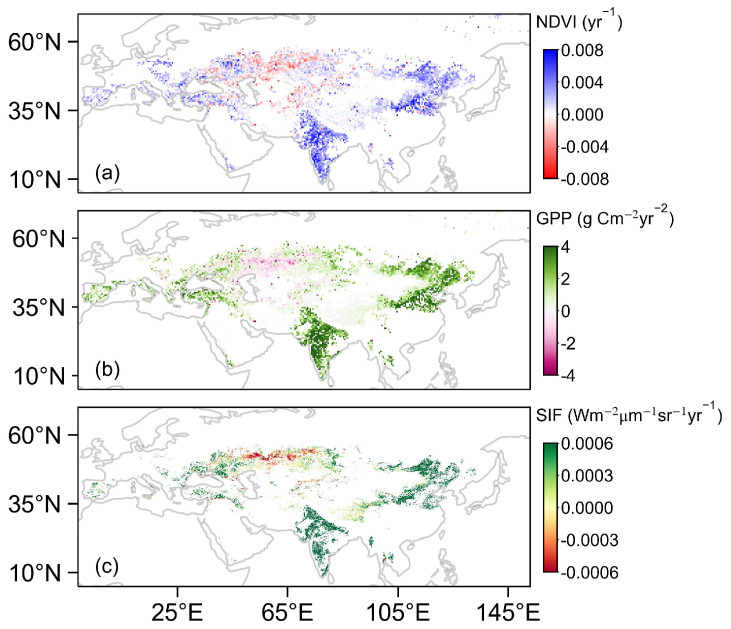
Spatial distribution of the temporal trends in normalized difference vegetation index (NDVI, (**a**)), gross primary productivity (GPP, (**b**)) and solar-induced chlorophyll fluorescence (SIF, (**c**)) during 2003–2022.

At the pixel scale, over the past two decades, the proportions of significant increases in NDVI, GPP and SIF in the Eurasian drylands were 29.22%, 36.97% and 41.90%, respectively (Figure 3). Spatially, except for the EEU (E. Europe), the increase in vegetation activity in ten sub-regions was consistent (Table 3). For NDVI, GPP and SIF, the increasing trend was the highest mainly in agricultural areas (Figure 1b), such as South Asia (SAS: 0.36 × 10^−2^ yr^−1^, 0.31 × 10^−2^ g C m^−2^ yr^−2^ and 0.17 × 10^−2^ Wm^−2^µm^−1^sr^−1^yr^−1^) and East Asia (EAS: 0.29 × 10^−2^ yr^−1^, 0. 29 × 10^−2^ g C m^−2^ yr^−2^ and 0.16 × 10^−2^ Wm^−2^µm^−1^sr^−1^yr^−1^). In contrast, for NDVI, GPP and SIF, only 2.31%, 1.19% and 2.92% decreased significantly (*p* < 0.05); the trend in EEU was the lowest (−0.02 × 10^−2^ yr^−1^, 0.01 × 10^−2^ g C m^−2^ yr^−2^ and 0.003 × 10^−2^ Wm^−2^µm^−1^sr^−1^yr^−1^) (Table 3).

### 3.2. Spatiotemporal Patterns of Natural Factors in Eurasian Drylands

**Figure 4:**
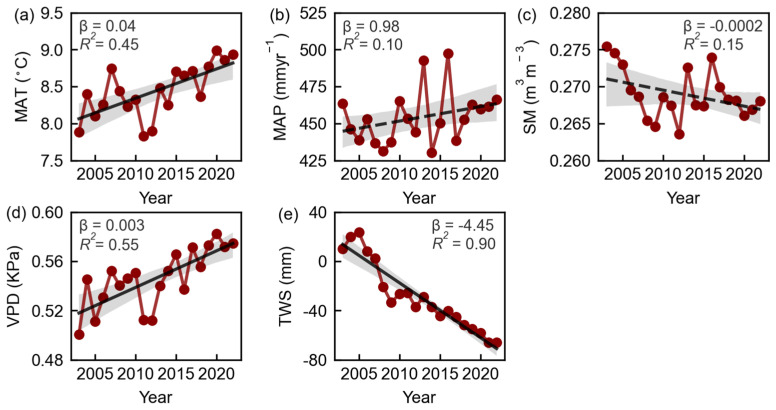
Temporal trends in the mean annual temperature (MAT, (**a**)), mean annual precipitation (MAP, (**b**)), soil moisture (SM, (**c**)), vapor pressure deficit (VPD, (**d**)) and terrestrial water storage (TWS, (**e**)) in Eurasian drylands during 2003–2022, respectively. Solid (dashed) lines indicate significant (insignificant) regressions (*p* < 0.05, the Student’s *t*-test).

From 2003 to 2022, the mean annual temperature (MAT) and atmospheric vapor pressure deficit (VPD) of the Eurasian drylands significantly increased at rates of 0.04 °C yr^−1^ (*p* < 0.01) and 0.003 K Pa yr^−1^ (*p* < 0.01), respectively (Figure 4a,d). The trends in soil moisture (SM) and mean annual precipitation (MAP) did not pass statistical significance tests (*p* > 0.05) (Figure 4b,c). In contrast, terrestrial water storage (TWS) experienced significant decreases at rates of 4.45 mm yr^−1^ (*p* < 0.01) (Figure 4e).

**Figure 5:**
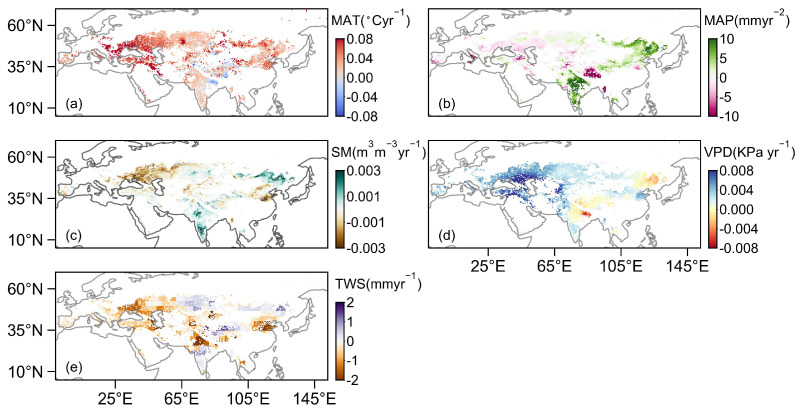
Spatial distribution of the linear trends in mean annual temperature (MAT, (**a**)), mean annual precipitation (MAP, (**b**)), soil moisture (SM, (**c**)), vapor pressure deficit (VPD, (**d**)) and terrestrial water storage (TWS, (**e**)) from 2003 to 2022.

Spatially, from 2003 to 2022, over 38.88 % of the Eurasian drylands exhibited a significant increasing trend in MAT (Figure 5a), with particularly notable increases in WCE (West and Central Europe), EEU (E. Europe), the MED (Mediterranean), WCA (W. C. Asia) and ARP (Arabian Peninsula) (Table 4). In these regions, MAT increased at a rate higher than 0.05 °C yr^−1^ (Table 4). MAP increased in more than 58.24% of the Eurasian drylands (Figure 5b), primarily located in ESB (E. Siberia: 4.82 mm yr^−2^) and SAS (S. Asia: 6.97 mm yr^−2^) (Table 4). However, regions exhibiting a decreasing trend in MAP were primarily situated in WCE (−1.49 mm yr^−2^), EEU (−0.79 mm yr^−2^), the MED (−0.79 mm yr^−2^), WCA (−1.47 mm yr^−2^) and TIB (Tibet Plateau: −3.88 mm yr^−2^) (Table 4). Except for ESB, TIB and SAS, 57.71% of the Eurasian drylands experienced a decreasing trend in SM over this period (Figure 5c). Meanwhile, VPD significantly increased in 47.96% of the Eurasian drylands (Figure 5d). In WCE (0.005 K Pa yr^−1^), EEU (0.006 K Pa yr^−1^), the MED (0.005 K Pa yr^−1^) and WCA (0.006 K Pa yr^−1^) (Table 4), the increasing trend in VPD was higher than in other regions. The decreasing trend in TWS was significant in the Eurasian drylands (55.80%) (Figure 5e), mainly concentrated in the WCE (−1.05 mm yr^−1^), WCA (−0.88 mm yr^−1^) and EAS (E. Asia: −0.84 mm yr^−1^).

### 3.3. Response Characteristics of NDVI, GPP and SIF to Natural Factors in Eurasian Drylands

**Figure 6:**
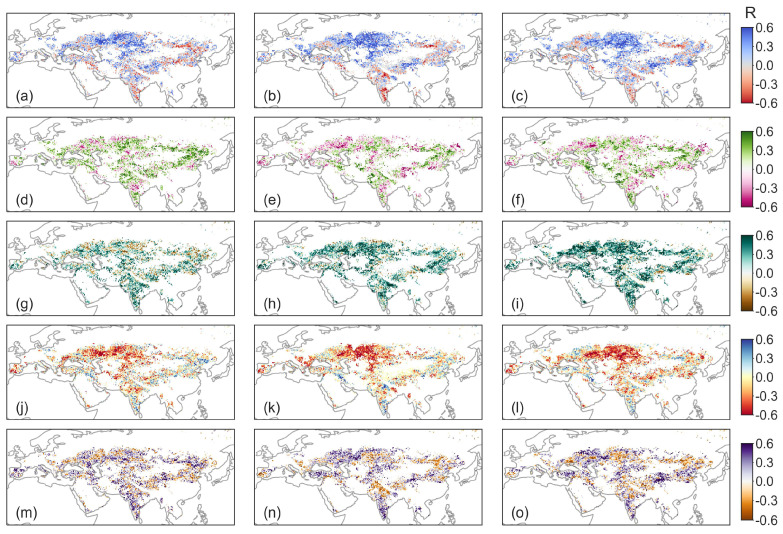
Spatial patterns of partial correlation coefficient between NDVI, GPP, SIF and natural factors. (**a**,**d**,**g**,**j**,**m**) show the partial correlation coefficient between normalized difference vegetation index (NDVI) and mean annual temperature (MAT), mean annual precipitation (MAP), soil moisture (SM), vapor pressure deficit (VPD) and terrestrial water storage (TWS), respectively. (**b**,**e**,**h**,**k**,**n**) show the partial correlation coefficient between gross primary productivity (GPP) and MAT, MAP, SM, VPD and TWS, respectively. (**c**,**f**,**i**,**l**,**o**) show the partial correlation coefficient between solar-induced chlorophyll fluorescence (SIF) and MAT, MAP, SM, VPD and TWS, respectively.

To reveal the impact of natural factors on vegetation temporal variability, we conducted partial correlation analyses between detrended vegetation activity factors (normalized vegetation index (NDVI), gross primary productivity (GPP) and solar-induced chlorophyll fluorescence (SIF)) and natural factors (mean annual temperature (MAT), mean annual precipitation (MAP), soil moisture (SM), atmospheric vapor pressure deficit (VPD) and terrestrial water storage (TWS)). We found that the partial correlation coefficients between the three indicators representing vegetation activity, namely NDVI, GPP and SIF, exhibited similar spatial patterns with natural factors (Figure 6). In summary, the interannual variability of vegetation temporal variability showed a stronger correlation with changes in SM compared to MAT, MAP, VPD and TWS (Figure 6). The positive influence of SM on SIF (33.47%) was stronger compared to its influence on the NDVI (14.23%) or GPP (21.65%) (Figure 6g–i). Additionally, VPD had a significantly negative impact on SIF (14.23%) compared to its impact on NDVI (11.27%) or GPP (12.73%) (*p* < 0.05) (Figure 6j–l).

**Figure 7:**
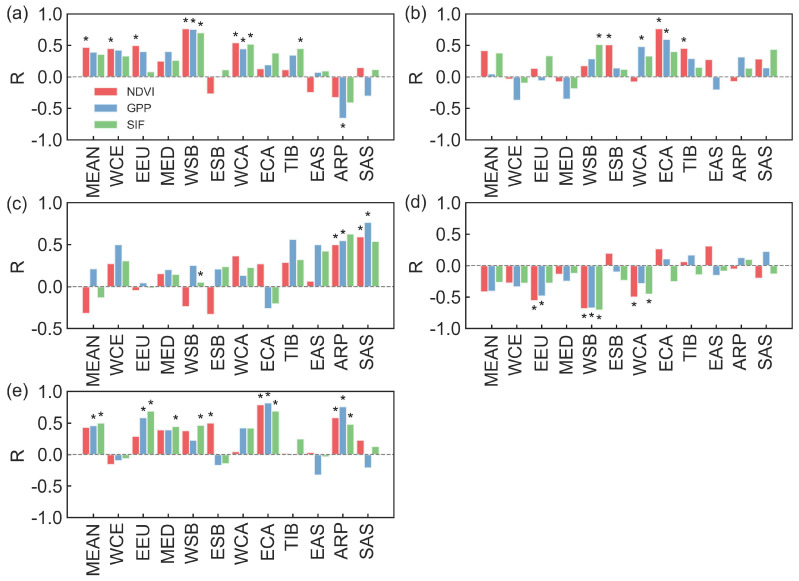
Partial correlation coefficient between NDVI, GPP, SIF and natural factors. Red, blue and green bars represent the normalized difference vegetation index (NDVI), the gross primary productivity (GPP) and the solar-induced chlorophyll fluorescence (SIF), respectively. MAT (**a**), MAP (**b**), SM (**c**), VPD (**d**) and TWS (**e**) represent the mean annual temperature, the mean annual precipitation, the soil moisture, the vapor pressure deficit and the terrestrial water storage, respectively. WCE, EEU, the MED, WSB, ESB, WCA, ECA, TIB, EAS, ARP and SAS represent West and Central Europe, E. Europe, Mediterranean, W. Siberia, E. Siberia, W. C. Asia, E. C. Asia, Tibet Plateau, E. Asia, Arabian Peninsula and S. Asia, respectively. The symbol “*” indicates that the partial correlation coefficient has passed the significance test with *p* < 0.05.

Regions with a position relationship MAT with vegetation temporal variability (NDVI, GPP and SIF) were located in WSB (W. Siberia) (Figure 7a). Meanwhile, the strongest negative impacts of VPD on vegetation temporal variability (NDVI, GPP and SIF) were mainly distributed in WSB (Figure 7d). The positive impacts of MAP on NDVI, GPP and SIF were in WSB, ESB (E. Siberia), ECA (E. C. Asia) and TIB (Tibet Plateau); however, the results presented the opposite findings in WCE (West and Central Europe) and the MED (Mediterranean) (Figure 7b). The positive impacts of SM on NDVI, GPP and SIF in WCE, the MED, WCA (W. C. Asia), TIB, EAS (E. Asia), ARP (Arabian Peninsula) and SAS (S. Asia) were found. Opposite findings were found in EEU (E. Europe), WSB and ESB for NDVI (Figure 7c). In regions where TWS is positively correlated with vegetation temporal variability (NDVI, GPP and SIF) are mainly distributed in the EEU, the MED, WSB, ECA and ARP, the correlation of TWS is lower in WCE (Figure 7e).

### 3.4. Relative Contributions of Natural and Human Factors to Vegetation Activities

**Figure 8:**
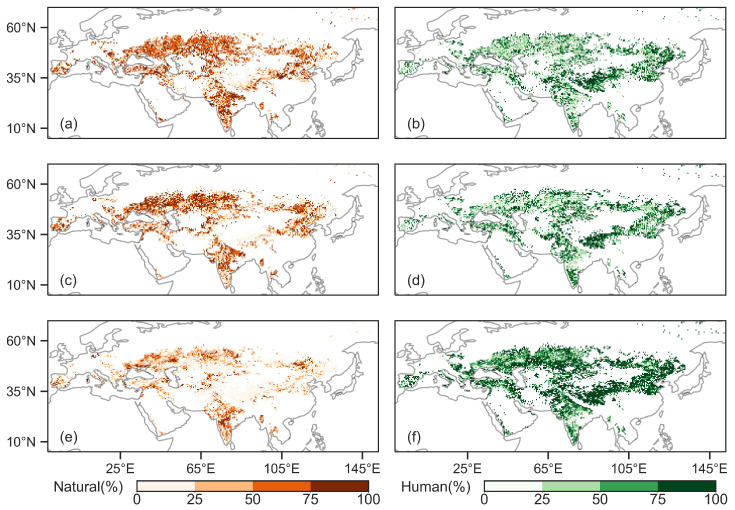
Spatial distributions of the relative contributions of natural and human factors to the changes in the normalized difference vegetation index (NDVI, (**a**,**b**)), gross primary productivity (GPP, (**c**,**d**)) and solar-induced chlorophyll fluorescence (SIF, (**e**,**f**)). Left and right columns represent natural and human factors, respectively.

Figure 8 illustrates the spatial distribution of the relative contributions from natural factors (mean annual temperature (MAT), mean annual precipitation (MAP), soil moisture (SM), atmospheric vapor pressure deficit (VPD) and terrestrial water storage (TWS)) and human activities to the interannual variations of the normalized vegetation index (NDVI), gross primary productivity (GPP) and solar-induced chlorophyll fluorescence (SIF) during 2003–2022. We found that the impact of human activities on vegetation greening was generally greater than that of the natural factors. Overall, the relative contributions of human-induced changes to NDVI, GPP and SIF were 52.45%, 55.81% and 74.18%, respectively, while the relative contributions of natural factors were 47.55%, 44.19% and 25.82%, respectively (Figure 8).

**Figure 9:**
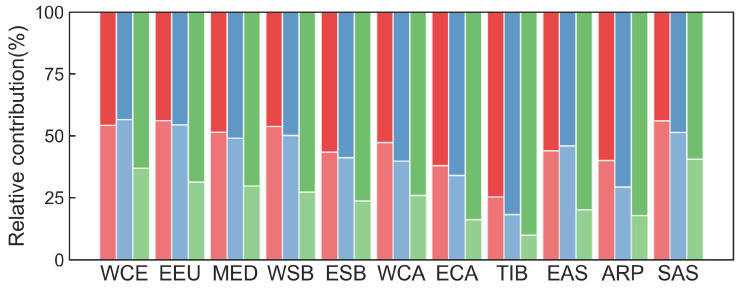
Relative contributions of natural and human factors to the changes in the normalized difference vegetation index (NDVI), gross primary productivity (GPP) and solar-induced chlorophyll fluorescence (SIF) in WCE (West and Central Europe), EEU (E. Europe), the MED (Mediterranean), WSB (W. Siberia), ESB (E. Siberia), WCA (W. C. Asia), ECA (E. C. Asia), TIB (Tibet Plateau), EAS (E. Asia), ARP (Arabian Peninsula) and SAS (S. Asia). Red, blue and green bars represent NDVI, GPP and SIF, respectively.

At the regional scale, human activities dominated the change in NDVI in most sub-regions, being the highest of relative contribution in TIB (Tibet Plateau: 74.68%), followed by ECA (E. C. Asia: 61.99%) and the largest contribution from natural factors was EEU (E. Europe: 56.14%) (Figure 9). For GPP, the relative contribution of natural factors was higher than that of human activities in WCE (West and Central Europe: 56.64% vs. 43.36%) and EEU (54.56% vs. 45.44%). For ESB (E. Siberia), WCA (W. C. Asia), ECA, TIB, EAS (E. Asia) and ARP (Arabian Peninsula), the relative contribution of human activities were greater than the relative contribution of natural factors on GPP (58.78%, 60.16%, 65.97%, 81.77%, 54.03% and 70.72%, respectively). The relative contributions of natural factors and human activities to the increase in GPP were counterbalanced in the MED (Mediterranean: 49.09% vs. 50.91%), WSB (W. Siberia: 50.15% vs. 49.85%) and SAS (S. Asia: 51.43% vs. 48.57%) (Figure 9). For SIF, the contribution of human activities was higher than 62.00% in eleven sub-regions, being the highest in TIB (90.15%) during 2003–2022 (Figure 9).

**Figure 10:**
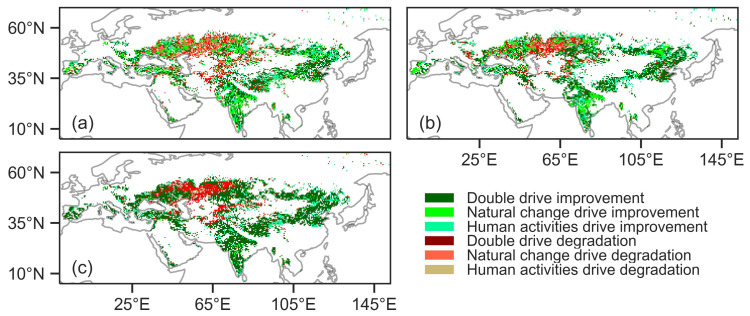
Spatial distribution of normalized difference vegetation index (NDVI, (**a**)), gross primary productivity (GPP, (**b**)) and solar-induced chlorophyll fluorescence (SIF, (**c**)) change drivers. “Improvement” represents an increasing trend in vegetation index over the past 20 years, while “degradation” indicates a declining trend in vegetation index over the same period.

Further analysis of the improved (i.e., the index values show an increasing trend) regions for NDVI, GPP and SIF indicated that the total contributions of human and natural factors were 30.38%, 31.68% and 42.88%, respectively (Figure 10). In the Eurasian drylands, human activity predominated, accounting for 23.20%, 28.50% and 21.10% of NDVI-, GPP- and SIF-increased areas. Natural factors, on the other hand, dominated, constituting 15.97%, 13.09% and 2.51% of NDVI-, GPP-, SIF-increased areas. In areas where NDVI, GPP and SIF were declining, indicating vegetation degradation, the relative contributions of human and natural factors were 12.20%, 6.86% and 19.17%, respectively. Human activity predominated, accounting for 10.87%, 6.92% and 12.85% of NDVI-, GPP- and SIF-decreased areas. On the other hand, natural factors dominated, constituting 7.37%, 2.95% and 1.50% of NDVI-, GPP-, SIF-decreased areas (Figure 10).

We further quantified the contribution of human activities and natural factors to vegetation in both grassland and cropland (Table 5). In cropland with increasing vegetation, the contribution of human activities to vegetation was greater than that of natural factors (NDVI: 9.92% > 8.69%, GPP: 12.55% > 9.94% and SIF: 10.62% > 1.18%). In cropland with vegetation degradation, for NDVI and GPP, the contribution of natural factors to vegetation was higher than that of human activities (NDVI: 11.29% > 6.33% and GPP: 6.90% > 4.62%). However, for SIF, human activities had a greater impact (SIF: 2.97% > 0.88%). In grassland regions with increasing vegetation, the contribution of human activities was higher than that of natural factors (NDVI: 10.07% > 5.49%, GPP: 14.35% > 3.93% and SIF: 8.96% > 0.24%). In regions with vegetation degradation, for NDVI and GPP, the contributions of human activities and natural factors were consistent (NDVI: 8.85% vs. 8.38% and GPP: 4.43% vs. 4.58%). However, for SIF, human activities contributed more than natural factors (SIF: 10.69% > 0.40%).

## 4. Discussion

In recent decades, vegetation activity in the Eurasian drylands has undergone significant changes driven by natural factors such as temperature, precipitation, soil moisture, atmospheric vapor pressure deficit and terrestrial water storage [6,56,57]. The prominent role of the Eurasian drylands in the global ecosystem necessitates a more in-depth understanding of the changing trends in vegetation activity in this region under the backdrop of global natural change [6,30]. This study, combining various indices reflecting vegetation activity, comprehensively analyzed the changing trends in vegetation greenness (represented by NDVI), productivity (represented by GPP) and photosynthetic capacity (represented by SIF) in the Eurasian drylands over the past two decades. Consistent with previous research results, overall, vegetation greenness and productivity in the Eurasian drylands show a significant increasing trend [4]. Furthermore, this study reveals that the photosynthetic capacity of vegetation in the Eurasian drylands generally exhibits a significant upward trend, with the proportion of areas showing a significant increase reaching 41.90%. Additionally, the study unveils a distinct spatial pattern in the changing trends in vegetation activity in the Eurasian drylands, with South Asia, East Asia, West Siberia and the Mediterranean region experiencing a much higher degree of enhancement in vegetation activity compared to other regions. In contrast, Central Asia showed trends in browning, which is similar to the results of Zhang et al. [58]. The spatial differences emphasize the importance of considering regional-specific factors when assessing vegetation activity and analyzing influencing factors in the Eurasian drylands.

In this study, the impact of five natural factors, including temperature, precipitation, soil drought, atmospheric drought and terrestrial water storage, on vegetation activity in the Eurasian drylands was quantified, with a further analysis of their regional specificity. Previous studies have indicated that the vegetation variability in the Eurasian drylands are primarily influenced by MAP, whereby an increase in MAP can improve SM and promote vegetation greening [30,59,60]. However, Ukkola et al. [60] reported that precipitation explains variability in dryland vegetation greenness globally but not locally. Liu et al. [30] argued that the dynamics of global dryland vegetation were more sensitive to soil moisture. This study validates this point and reveals that, compared to atmospheric drought and terrestrial water storage, soil moisture has a greater impact on vegetation activity in the Eurasian drylands. The shallow root systems of vegetation greening (grassland and cropland) make them more susceptible to changes in available water. Generally, a higher VPD and lower water availability (SM and TWS) would lead to a reduction in NDVI, GPP and SIF. However, the widespread intensified MAT and VPD and decreased water availability (SM and TWS) in Eurasia resulted in increases in NDVI, GPP and SIF. Our results reported that dryland vegetation was dominated by soil moisture in Eurasia. Liu et al. [30] also described that the global drylands have become greener and more productive, albeit with an enhanced VPD. In West Siberia (WSB), the effect of MAT and VPD on vegetation greening was higher than that of SM, indicating that vegetation activity in high latitudes is highly temperature-driven. Zhong et al. [61] reported that the interaction between VPD and T/SM not only reduced the enhanced vegetation productivity due to warming but also increased soil drought. While TWS comprises soil moisture, surface waters and groundwater, the deep groundwater level in the Eurasian drylands makes vegetation activity more sensitive to soil moisture than to TWS [62,63,64]. Consistent with previous research findings, this study also revealed that human activities have played a dominant role in driving changes in vegetation greening in the Eurasian drylands [65].

However, the contribution of human activities to vegetation greening varies across different regions. Among them, human activities have the highest contribution to East Siberia, West and Central Asia, West and Central Asia, Tibet Plateau, East Asia, and the Arabian Peninsula, possibly linked to national policies determining regional vegetation types [66]. For example, since the late 1970s, China has enacted 13 national initiatives aimed at restoring grassland and cropland [34]. Yan et al. [67] found that grassland productivity increased due to the implementation of the Grain for Green Program (GGP) in Shaanxi Province. Zhang et al. [68] reported that human activities led to the grassland degradation in Inner Mongolia, but that the grassland protection policies and ecological restoration projects of the Chinese government improved actual net primary productivity in most of the region’s grassland from 2001 to 2014. Pei et al. [69] noted that human activities contributed to 66% of the NDVI increase in the agro-pastoral ecotone of northern China.

However, we found that human activities are also a significant factor contributing to vegetation degradation in certain regions. In many areas of Kazakhstan, vegetation greenness shows a declining trend, possibly linked to the conversion of extensive grassland in the northern part of Kazakhstan into farmland during the Soviet era, with many of these farmlands abandoned after the dissolution of the Soviet Union. Despite ecological conservation measures such as fencing and rotational grazing, the rapid increase in livestock numbers may exacerbate grazing pressure, leading to intensified vegetation degradation [5]. Similarly, in some areas of the Loess Plateau, vegetation restoration plans may inadvertently result in vegetation degradation due to water demand surpassing the regional water resource-carrying capacity [36]. Additionally, rapid population growth and fast urbanization may contribute to vegetation reduction in certain regions [70]. Therefore, the any ecological restoration policy that will be formulated needs to consider both natural changes and the actual geographical environment.

While this study quantifies the impact of natural and human factors on vegetation activity in the Eurasian drylands, there is still a certain degree of uncertainty. For example, we analyzed the correlation between temperature, precipitation, soil moisture, atmospheric drought and terrestrial water storage with vegetation activity, without considering the impact of factors such as CO_2_ concentration, wind speed, relative humidity and solar radiation [71]. Therefore, the results of this study may somewhat overestimate the impact of human activities. For instance, the increase in CO_2_ concentration can reduce stomatal conductance, enhance water use efficiency and decrease plant water demand, thereby influencing vegetation activity [72]. Additionally, factors such as the low resolution of remote sensing and land-use cover data, the low sensitivity of vegetation indices in areas with low vegetation cover and potential cumulative and lag effects of climate factors on vegetation activity could all have an impact on our study results, necessitating further refinement in future research.

## 5. Conclusions

Against the backdrop of rising temperatures, decreasing water supply and increasing human activities, gaining an understanding of the changing characteristics of vegetation activity in the Eurasian drylands and its response to natural and human-driven factors holds significant scientific value. This study comprehensively analyzed the changing characteristics of vegetation greenness, productivity and photosynthetic capacity in the Eurasian drylands over the past two decades and systematically quantified the impact of various natural factors and human activities on vegetation activity. We found that, over the regional scale, between 2003 and 2022, vegetation activity in the Eurasian drylands exhibited an enhancing trend. The growth rates of the normalized vegetation index (NDVI), gross primary productivity (GPP) and solar-induced chlorophyll fluorescence (SIF) in the the Eurasian drylands were 1.00 × 10^−3^ y^−1^ (*p* < 0.01), 1.30 g C m^−2^ y^−2^ (*p* < 0.01) and 1.00 × 10^−3^ W m^−2^µm^−1^sr^−1^yr^−1^ (*p* < 0.01), respectively. At the pixel scale, regions with significantly increased vegetation greenness, productivity and photosynthetic capacity accounted for 29.22%, 36.97% and 41.90% of the Eurasian drylands, respectively. In terms of spatial patterns, the most pronounced enhancements in vegetation activity were observed in South Asia, East Asia, Central Europe and the Mediterranean region. Additionally, we found that the correlation between soil moisture (SM) and vegetation temporal variability in the Eurasian drylands surpassed that of the mean annual temperature (MAT), the mean annual precipitation (MAP), the atmospheric vapor pressure deficit (VPD) and the terrestrial water storage (TWS), highlighting the pivotal role of soil moisture in influencing vegetation temporal variability. Human activities emerged as the predominant driving factors for vegetation greening in the Eurasian drylands, with average relative contributions to NDVI, GPP and SIF of 52.45%, 55.81% and 74.18%, respectively. Our findings enrich the current understanding of the complex vegetation-natural relationships in the Eurasian drylands and emphasize the importance of considering human-induced factors in future research on the driving factors of vegetation activity.

## Figures and Tables

**Table 1:** The LC types analyzed in this research and the original ESA-CCI-LC classes.

LC Types Used in This Study	Original LC Class Codes in the ESA-CCI-LC Dataset Combined to Form the LC Types Used in This Study	Description of the Original LC Classes in the ESACCI-LC Dataset
Cropland	10, 11, 12	Rainfed cropland
	20	Irrigated cropland
	30	Mosaic cropland (>50%)/natural vegetation (tree, shrub, herbaceous cover) (<50%)
	40	Mosaic natural vegetation (tree, shrub, herbaceous cover) (>50%)/cropland (<50%)
Grassland	130	Grassland
	150	Sparse vegetation

**Table 2:** The relative contributions of natural change and human activities.

Vegetation Trend	Predicted and Residual		Relative Contributions (%)		Explanation
	${slope}_{pre}$	${slope}_{pre}$	Natural	Human	
Improvement (${slope}_{obs}$ > 0)	>0	>0	$\frac{{slope}_{pre}}{{slope}_{pre}+{slope}_{res}}\times 100$	$\frac{{slope}_{res}}{{slope}_{pre}+{slope}_{res}}\times 100$	Double
	>0	<0	100	0	Natural
	<0	>0	0	100	Human
Degradation (${slope}_{obs}$ < 0)	<0	<0	$\frac{{slope}_{pre}}{{slope}_{obs}}\times 100$	$\frac{{slope}_{res}}{{slope}_{obs}}\times 100$	Double
	<0	>0	100	0	Natural
	>0	<0	0	100	Human

**Table 3:** The linear regression coefficients for normalized difference vegetation index (NDVI), gross primary productivity (GPP) and solar-induced chlorophyll fluorescence (SIF) during 2003–2022 in WCE (West and Central Europe), EEU (E. Europe), ME (Mediterranean), WSB (W. Siberia), ESB (E. Siberia), WCA (W. C. Asia), ECA (E. C. Asia), TIB (Tibet Plateau), EAS (E. Asia), ARP (Arabian Peninsula) and SAS (S. Asia). The symbol “*” indicates that the linear regression coefficient has passed the significance test with *p* < 0.05 (the Student’s *t*-test).

Variables	NDVI (10^−2^ yr^−1^)	GPP (10^−2^ g C m^−2^ yr^−2^)	LAI (10^−2^ Wm^−2^µm^−1^sr^−1^yr^−1^)
WCE	0.12 *	0.06	0.06 *
EEU	−0.02	0.01	0.003
MED	0.16 *	0.16 *	0.09 *
WSB	0.01	0.05	0.02
ESB	0.19 *	0.20 *	0.09 *
WCA	0.05	0.06 *	0.03 *
ECA	0.12 *	0.08 *	0.04 *
TIB	0.07 *	0.05 *	0.03 *
EAS	0.29 *	0.29 *	0.16 *
ARP	0.10 *	0.06 *	0.05 *
SAS	0.36 *	0.31 *	0.17 *

**Table 4:** The linear regression coefficients for mean annual temperature (MAT), mean annual precipitation (MAP), soil moisture (SM), vapor pressure deficit (VPD) and terrestrial water storage (TWS) during 2003-2022 in WCE (West and Central Europe), EEU (E. Europe), MED (Mediterranean), WSB (W. Siberia), ESB (E. Siberia), WCA (W. C. Asia), ECA (E. C. Asia), TIB (Tibet Plateau), EAS (E. Asia), ARP (Arabian Peninsula) and SAS (S. Asia). The symbol “*” indicates that the linear regression coefficient has passed the significance test with *p* < 0.05 (the Student’s *t*-test).

Variables	MAT (°C yr^−1^)	MAP (mm yr^−2^)	SM (m^3^ m^−3^ yr^−1^)	VPD (K Pa yr^−1^)	TWS (mm yr^−1^)
WCE	0.07 *	−1.49	−0.0012 *	0.005 *	−1.05 *
EEU	0.05 *	−0.79	−0.0014 *	0.006 *	−0.67 *
MED	0.06 *	−0.79	−0.0004	0.005 *	−0.52 *
WSB	0.03	0.47	−0.0002	0.003 *	0.12
ESB	0.04	4.82 *	0.0008 *	0.001	0.19 *
WCA	0.05 *	−1.47	−0.0004	0.006 *	−0.88 *
ECA	0.03	0.76	−0.0003	0.002 *	−0.46 *
TIB	0.01	−3.88	0.0002	−0.000	−0.16 *
EAS	0.04 *	3.37	−0.0006	0.001	−0.84 *
ARP	0.06 *	0.44	−0.0002	0.004 *	−0.37 *
SAS	0.01	6.97 *	0.0008 *	0.001	−0.55 *

**Table 5:** The pixel ratio of driving factors. “Improvement” represents an increasing trend in vegetation over the past 20 years, while “Degradation” indicates a declining trend in vegetation over the same period. “Double” is the contributions of natural and human factors to vegetation, “natural” represents the contributions of natural to vegetation, and “human” indicates the contributions of human to vegetation.

Variables			Cropland (%)	Grassland (%)
NDVI	Improvement	double	7.42	6.46
		natural	8.69	5.49
		human	9.92	10.07
	Degradation	double	8.40	8.71
		natural	11.29	8.85
		human	6.33	8.38
GPP	Improvement	double	11.22	9.30
		natural	9.94	3.93
		human	12.55	14.35
	Degradation	double	9.77	8.41
		natural	s6.90	4.43
		human	4.62	4.58
SIF	Improvement	double	18.02	14.72
		natural	1.18	0.24
		human	10.62	8.96
	Degradation	double	16.07	15.24
		natural	0.88	0.40
		human	2.97	10.69

## Data Availability

The data used in the present work have been listed in Data Sources.
